# Little effects of Insulin-like Growth Factor-I on testicular atrophy induced by hypoxia

**DOI:** 10.1186/1471-2490-6-4

**Published:** 2006-02-21

**Authors:** Fernando Diez-Caballero, Inma Castilla-Cortázar, Maria Garcia-Fernandez, Juan Enrique Puche, Matias Diaz-Sanchez, Amelia Diaz Casares, M Aurelia Aliaga-Montilla, Coronación Rodriguez-Borrajo, Salvador Gonzalez-Barón

**Affiliations:** 1 Departments of Physiology and Urology. University of Navarra. Pamplona, Spain; 2 Department of Human Physiology. School of Medicine. University of Málaga, Spain; 3 Department of Human Physiology. School of Medicine. University San Pablo-CEU, Spain

## Abstract

**Background:**

Insulin-like Growth Factor-I (IGF-I) supplementation restores testicular atrophy associated with advanced liver cirrhosis that is a condition of IGF-I deficiency. The aim of this work was to evaluate the effect of IGF-I in rats with ischemia-induced testicular atrophy (AT) without liver disease and consequently with normal serum level of IGF-I.

**Methods:**

Testicular atrophy was induced by epinephrine (1, 2 mg/Kg intra-scrotal injection five times per week) during 11 weeks. Then, rats with testicular atrophy (AT) were divided into two groups (n = 10 each): untreated rats (AT) receiving saline sc, and AT+IGF, which were treated with IGF-I (2 μg.100 g b.w.^-1^.day^-1^, sc.) for 28d. Healthy controls (CO, n = 10) were studied in parallel. Animals were sacrificed on day 29^th^. Hypophyso-gonadal axis, IGF-I and IGFBPs levels, testicular morphometry and histopathology, immuno-histochemical studies and antioxidant enzyme activity phospholipid hydroperoxide glutathione peroxidase (PHGPx) were assessed.

**Results:**

Compared to controls, AT rats displayed a reduction in testicular size and weight, with histological testicular atrophy, decreased cellular proliferation and transferrin expression, and all of these alterations were slightly improved by IGF-I at low doses. IGF-I therapy increased signifincantly steroidogenesis and PHGPx activity (p < 0.05). Interestingly, plasma IGF-I did not augment in rats with testicular atrophy treated with IGF-I, while IGFBP3 levels, that reduces IGF-I availability, was increased in this group (p < 0.05).

**Conclusion:**

In testicular atrophy by hypoxia, condition without IGF-I deficiency, IGF-treatment induces only partial effects. These findings suggest that IGF-I therapy appears as an appropriate treatment in hypogonadism only when this is associated to conditions of IGF-I deficiency (such as Laron Syndrom or liver cirrhosis).

## Background

Insulin-Like Growth Factor-I (IGF-I) is an anabolic hormone, produced mainly in the liver by GH stimulation [1]. In advanced liver cirrhosis plasma levels of IGF-I are reduced [1] because liver biosynthesis and GH receptor expression in hepatocytes are decreased. Testicular atrophy is a common complication in advanced cirrhosis. Previous results have shown that IGF-I supplementation recovers testicular atrophy associated to experimental cirrhosis [1]. Since advanced cirrhosis is a condition of "IGF-I deficiency" and IGF-I therapy was able to revert testicular atrophy in cirrhotic rats in only three weeks, a direct effect of IGF-I on testes seems to be the most important factor to explain our findings. This idea is supported by the existence of receptors for IGF-I in Sertoli cells, germ cells and Leydig cells [2,3]. Following treatment with exogenous IGF-I patients with Laron dwarfism, a condition of IGF-I deficiency due to the absence of receptors for growth hormone, show an increase in testicular size and serum testosterone levels [4]. Stopping IGF-I administration led to a return of both parameters to the pretreatment situation indicating a specific effect of IGF-I [4].

On the other hand, since IGF-I therapy was also able to improve nutritional status, intestinal absorption and liver function tests [5-9], other factors could contribute to the gonadal improvement observed in these rats with cirrhosis treated with IGF-I.

The aims of the present study were: 1) to go more into the beneficial effects of the mechanisms mediated by IGF-I; and 2) investigate if IGF-I therapy could be an adequate treatment to improve testicular function in other conditions without liver disorder and consequently with normal serum levels of IGF-I.

With these objectives, the present work was carried out using an experimental model of hypoxia-induced testicular atrophy including three groups: healthy controls, untreated rats with testicular atrophy and rats with testicular atrophy treated with low doses of IGF-I during 28 days. Testes histopathology and function, pituitary-gonadal axis and IGF-I and IGFBPs plasma levels were assessed in the three experimental groups.

## Methods

### Induction of testicular atrophy

Testicular atrophy was induced as previously described [10]. Briefly, male Wistar rats (4 weeks old, 150–160 g) were subjected to 1.2 mg/Kg b.w. five times/week intra-scrotal injections of epinephrine in sterile saline (Sigma), for 11 weeks. Rats were housed in cages placed in a room with 12-hour light-dark cycle and constant humidity and temperature (20°C). Both, food (standard semipurified diet for rodents; B.K. Universal, Sant Vicent del Horts, Spain) and water were given *ad libitum*. Healthy age-matched control rats were studied in parallel. All experimental procedures were performed in conformity with The Guiding Principles for Research Involving Animals.

### Study design and IGF-I treatment

After testicular atrophy induction (after 11 weeks receiving epinephrine), rats with testicular atrophy were randomly assigned to receive either vehicle (saline) (Group AT, n = 10) or recombinant human IGF-I (Chiron) (2 μg × 100 g bw^-1^xday ^-1 ^in two divided doses, subcutaneously) (Group AT+IGF, n = 10) for four weeks. Control rats (Group CO, n = 10) received saline during the same period.

In the morning of day 0 (before treatment), animals were weighted and blood samples were drawn from the retroocular venous plexus from all rats with capillary tubes (Marienfeld, Germany). Serum samples were stored at -20°C until used for analytical purposes.

In the morning of the 29^th ^day, rats were weighted, blood was obtained again and processed as previously indicated and animals were sacrificed by decapitation. After the abdominal cavity was opened, the testes were dissected and weighted. The testicular diameters (AP and LM) were measured in testes previously fixed for histology, using a precision calliper, Mituyoto^® ^(± 0.05 mm).

### Testicular histopathology and PCNA and transferrin immunohisto-chemistry

Bouin-fixed tissues were processed and sections (4 μm) were stained with Haemotoxylin and Eosin and Masson's trichrome. For histopathological evaluation of testes 30 seminiferous tubules from each rat of the three groups were blindly evaluated by two observers and the arithmetic mean of the scores was taken as the final result. Transversal sections of seminiferous tubuli were examined and evaluation of histological changes was made using a light projection microscope (Micro Promar Leitz GMBH, Wetzlar, Germany) at 100 × magnification. The following parameters were studied: tubular diameter, quantitation of the presence of the different types of cells in tubuli, presence of peritubular fibrosis, and the number of proliferating cells. For general purposes Haematoxilin & Eosin and Masson's trichrome staining were used. Specific techniques for other purposes are specified in the corresponding paragraphs.

Tubular diameters were expressed in μm. Changes in tubuli were classified into five categories (Category I: highest damage to Category V: full normality). Category I: presence of only Sertoli cells; Category II: Sertoli cells plus spermatids, Category III: Sertoli cells, plus spermatides, plus spermatocytes, Category IV: presence of all kinds of cells but showing some morphological alterations (i.e.: severe vacuolization, aberrant cells). Category V: presence of all kinds of cells without morphological alterations. The presence of peritubular fibrosis was evaluated in Masson's trichrome preparations according to the thickness of the staining of collagen deposition surrounding tubuli. Proliferating cells were identified by immunostaining of proliferating cellular nuclear antigen (PCNA) using an avidin-biotin peroxidase method [11] with retrieval of antigen by means of microware irradiation. Specific anti PCNA antibody (mouse anti-PCNA, clone PC 10, DAKO, Denmark) biotinylated rabbit anti-mouse IgG (DAKO, Denmark) were used and the avidin-biotin complex technique (ABC, DAKO kit) was performed. The bound antibodies were visualized by means of 3,3'-diaminobenzidine tetrahydrochloride (SIGMA Chemical Company, St. Louis, MO) with nickel enhancement (Shu et al., 1988) [11]. Finally, samples were slightly counterstained (10 seconds) in hematoxilin, dehydrated, and mounted in DPX. Controls were performed by substitution of the primary antibody by TBS. The number of PCNA positive cells was recorded. The result was expressed as stained cells per tubuli (arithmetic mean of 30 screened tubuli).

Taking into account all the parameters specified above, an overall score of testicular histopathological damage was adopted according to the following guidelines: Tubular diameter (in μm) scored from 0 to 3 points: > 260 = 0 points; from 240 to 259 = 1 point; from 220 to 239 = 2 points; and < 219 = 3 points. Cellular counts in tubuli: Category I (8 points), category II (6 points), category III (4 points), category IV (2 points) and category V (0 points). The score was obtained by multiplying the number of tubuli in each category by its respective points divided by 30 (the number of tubuli evaluated in each animal). Peritubular fibrosis was scored from 0 (absent or minimal) to 1 (evident). Cellular proliferation (PCNA) was scored from 0 to 3 according to the following criteria: when the number of PCNA positive cells /tubule was higher than 60, the score was 0 point. When the number of PCNA positive cells / tubuli was lower than 60, the score was obtained according to the following formula: *(60 – PCNA positive cells) × 0.05*. Therefore the overall score of histopathological damage ranged from 0 (complete normality) to 15 (full abnormality)**.**

In addition, the expression of transferrin in tubuli was evaluated by immunostaining using similar technique as for PCNA with specific anti-transferrin antibody (obtained from rabbit, RARa/TRf, Nordic Immunological Laboratories, Teknovas, The Netherlands). Transferrin expression was scored from 0 to 4 points. If 30 tubuli expressed transferrin normally all over the germinal epithelium: 0 points. The remaining scores were obtained according to the following formula: *(30 - Tubuli showing expression of transferrin all over the germinal epithelium) × 0.075*.

### Analytical methods

Serum levels of albumin, total proteins, glucose, cholesterol and alkaline phosphatase, were determined by routine laboratory methods using a Hitachi 747 autoanalyzer (Boerhringer-Mannheim, Germany).

Serum levels of the different hormones were assessed by RIA in a GammaChen 9612 Plus (Serono Diagnostics, Roma, Italy) using specific commercial assay systems. The sensitivity (S) of total Testosterone assay was 4 ng/dL and the intraassay coefficient of variation (CV) was less than 7%. The sensitivity of free Testosterone assay was 0.15 pg/mL and the CV was <8%. The sensitivity of Estradiol was 7 pg/mL and the CV was <7% (Coat-a-Count, DPC (Diagnostic Products Corporation, Los Angeles, CA). The kits for Rat luteinizing Hormone (rLH) (S = 1.7 ng/mL and CV<10%) and Rat follicle stimulating hormone (rFSH) (S = 1.8 ng/mL and CV<6%) were provided from Amersham International plc (Little Chalfont Buckinghamshire, England HP7 9NA). Assessment of IGF-I was carried out after an alcohol extraction in order to eliminate IGFBPs (Nichols Institute Diagnostics, San Juan Capistrano, CA, USA). The sensitivity of this assay was 21 ng/mL and the intraassay coefficient of variation was less than 4%.

Antioxidant enzyme activity phospholipid hydroperoxide glutathione peroxidase was determined as previosuly reported [12]. Briefly, PHGPx catalizes the oxidation of reduced glutathione (GSH) by phospholipids hydroperoxide in presence of glutathione reductase and NADPH. Oxidized glutathione (GSSG) is immediately reduced to GSH with the oxidation of NADPH to NADP^+^. And the decrease of NADPH absorbance at 340 nm is assessed using a Cobas Mira autoanalyzer (ABXMicro, Germany). Finally, a unit of PHGPx was expressed as the amount of enzime required to oxide 1 μmol/min of NADPH at 37°C subtracting the blank of the reactives (S = 3.7 IU/L and CV<10%).

### Western-ligand blot

IGF binding proteins (IGFBPs) were studied using Western blot analysis which was performed as described by Hossenlopp et al [13] and Hardouin et al. [14]. Briefly, rat plasma (3 μL) was diluted up to 50 μL in 0.06 M Tris-HCl and 0.15 M NaCl, pH 6.8, with 5% SDS, 20% glycerol and 0.02% bromophenol blue. The solution was submitted to a 5–15% gradient SDS-PAGE. Proteins were blotted to a 0.2 μm nitrocellulose filter (Bio-Rad). Nitrocellulose filter was dried for 5 min at 37°C, followed by quenching firstly in Tris buffer alone, and then in Tris buffer with 3% NP-40 (30 min at 4°C). Finally, the filter was soaked in Tris buffer with 1% BSA and 0.1% Tween-20 for 60 min, and was then incubated over-night with 200000 cpm ^125^I-IGF-I. After washing several times with buffer, the filter was dried and, lastly, exposed to an autoradiographic film.

Densitographic quantitation of IGFBPs was expressed in arbitrary units of optic density (mm^2^)

### Statistical analysis

Data are expressed as mean (± SEM). To assess the homogeneity among the three groups of rats a Kruskall-Wallis test was used, followed by multiple post-hoc comparisons using the Mann-Whitney U test (two tailed) with Bonferroni adjustment. A regression model was fitted considering histopathological score, PCNA or transferrin expression scores and IGF-I plasma concentration as the dependent and independent variables respectively. Within groups differences between pre- and post-treatment values were assessed by means of Wilcoxon matched pairs signed rank sum test. Any p value less than 0.05 was considered to be statistically significant. Calculations were performed with SPSSWin v.6.0. Program.

## Results

At baseline, both groups with testicular atrophy showed similar serum levels of albumin, cholesterol, creatinine, alkaline phosphatase, glucose, total proteins and urea, which were all normal as compared with control rats (Table 1).

### Testicular morphology and morphometry

**Figure 1:**
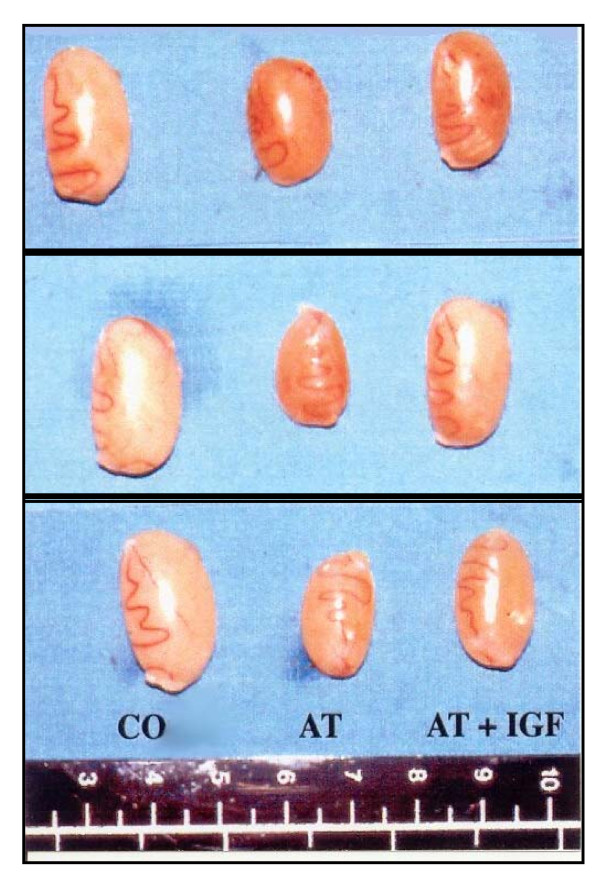
Macroscopic appearance of testes from healthy controls (CO), untreated rats with testicular atrophy (AT) and rats with testicular atrophy treated with IGF-I (AT+IGF). Note reduced volume of testes in AT rats.

There was a reduction in testicular size and volume in group AT as compared with control (Figure 1). Morphometric study showed a significant reduction in testicular weight (more expressive when values were corrected by body weight) in group AT compared to controls and AT+IGF group. Noteworthy, no significant differences were found between AT+IGF and controls. Longitudinal and transversal testicular diameters were reduced in AT group and increased significantly in rats treated with IGF-I (AT+IGF). Morphometric data in the three groups are summarized in Table 2.

### Testicular histopathology

**Figure 2:**
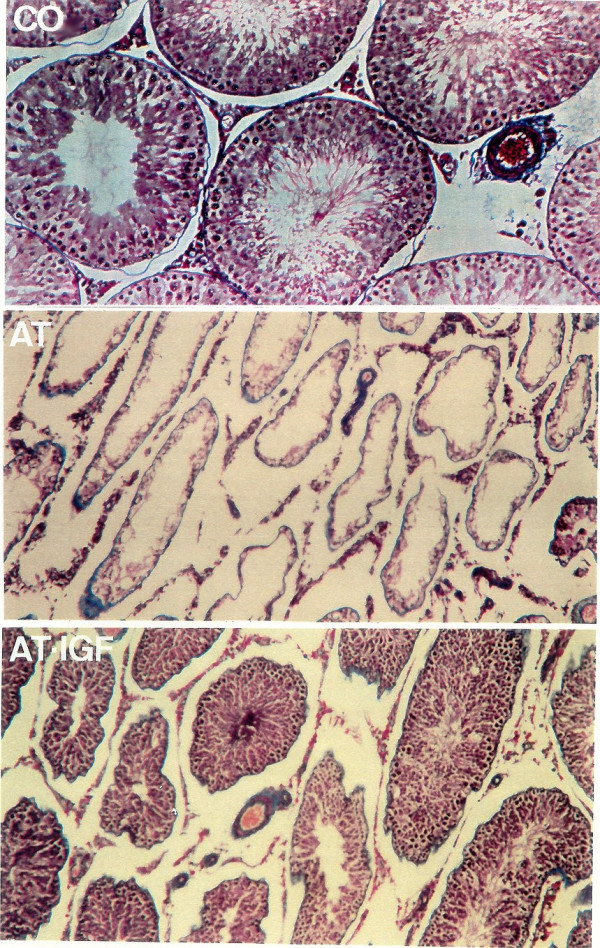
Microscopy of testes (× 100 magnification, Masson's stain). Testicular histological section of normal rat (CO) demonstrating active spermatogenesis in normal-size seminiferous tubuli with thin basement membranes and minimal peritubular fibrosis. Leydig cells are scarce, being widely separated by seminiferous tubuli. Seminiferous tubuli in testes from untreated animals with testicular atrophy (AT) appear seriously damaged. These animals show a decrease of tubular diameter, vacuolization in germinal epithelium, lost of germinal line, total reduction of spermatogenesis. AT rats treated with IGF-I (AT+IGF) show a significant improvement of all these alterations, although partial.

In AT group histological examination revealed testicular atrophy with marked decrease in tubular diameter expressed as an index of damage scored from 0 to 3 points (see Methods) (CO: 0.25 ± 0.05; AT: 2.07 ± 0.19, p < 0.001). IGF-I therapy induced a significant improvement of this parameter (1.45 ± 0.18, p < 0.05 vs group AT and p < 0.01 vs controls). Histopathological findings in untreated AT rats included: vacuolization of Sertoli's cells, loss of germinal line, detached germ cells, dramatic reduction of spermatogenesis, presence of abnormal spermatids or empty tubulli, and peritubular fibrosis. These alterations were much less intense or almost absent in rats receiving IGF-I (see Figure 2 for morphological comparison among groups). These changes are presented in Table 3 according to a pre-established score (see Methods), after exploring 30 tubuli from each preparation.

**Figure 3:**
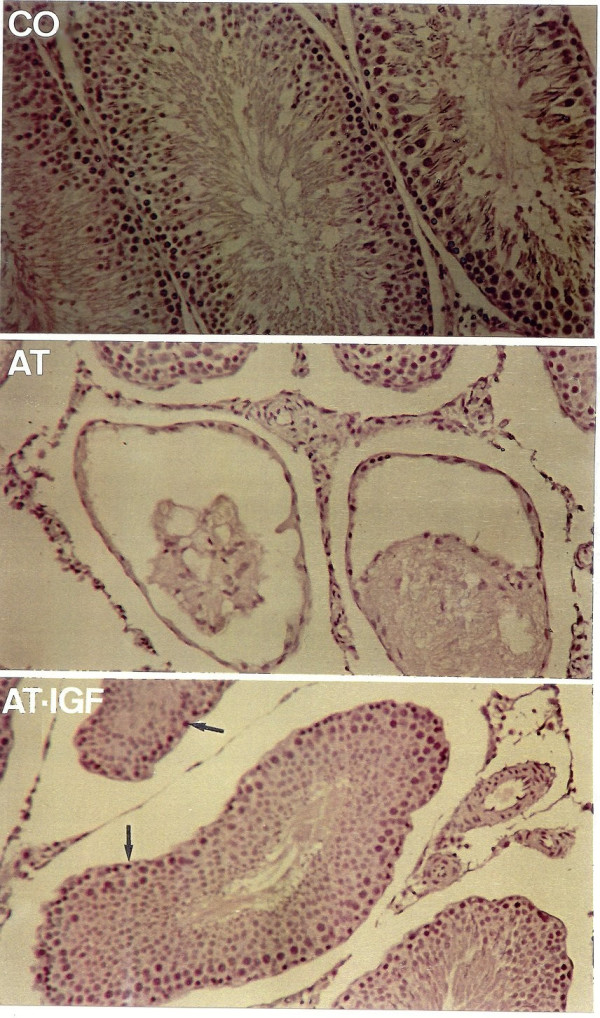
Study of proliferative activity, assessed by PCNA immunostaining. Reduced or absent cellular proliferation in untreated animals with testicular atrophy (AT) and a recovered germinal line in IGF-I-treated AT rats (AT+IGF) can be observed.

Testicular cellular proliferation, as evaluated by PCNA positive cells, was significantly reduced in both groups with testicular atrophy: IGF-I therapy induced an improvement but it did not reach statistical significance (mean of PCNA + cells scored in 30 tubuli for each animal; CO: 63.8 ± 1; AT: 36 ± 8, p < 0.001 AT vs CO; AT+IGF: 47 ± 5, p < 0.05 CO vs AT+IGF). Figure 3 shows PCNA immuno-histochemistry in the three groups.

Taking together all the morphological data a score of histopathological testicular damage was established (see Methods). The values of histopathological score were: CO: 0.49 ± 0.14; AT: 12.44 ± 1.29 (p < 0.001 AT vs CO); and AT+IGF: 7.78 ± 1.38 (p < 0.001 vs CO and p < 0.01 vs AT), demonstrating severe testicular damage in untreated AT rats and a significant improvement in AT rats treated with IGF-I.

Testicular transferrin, a marker of the integrity of the hemato-testicular barrier and Sertoli cell function, was evaluated from 0 to 4 points by immunohistochemistry in testicular slices (see Methods). Transferrin expression was decreased in AT rats (1.83 ± 0.43 points, p < 0.001 vs groups CO and AT+IGF) as compared with controls (3.85 ± 0.15 points) and to rats with testicular atrophy receiving IGF-I (2.60 ± 0.30 points, p < 0.001 vs groups CO and AT+IGF). A close inverse correlation was found between testicular transferrin expression and histopathological testicular damage score (r = -0.85, p < 0.001).

### Pituitary-gonadal axis

Untreated AT rats showed a significant reduction of serum levels of both total (p < 0.05) and free testosterone (p < 0.001) with increases of LH and FSH (p < 0.05) levels as well as the estradiol/testosterone ratio. However, in the group of rats with testicular atrophy treated for four weeks with low doses of IGF-I, a partial recovery was observed with an increase of the levels of both free (p < 0.05 vs untreated group) and total testosterone (p = 0.06 vs untreated group) and a significant reduction of the estradiol/total testosterone ratio (Table 4).

**Figure 4:**
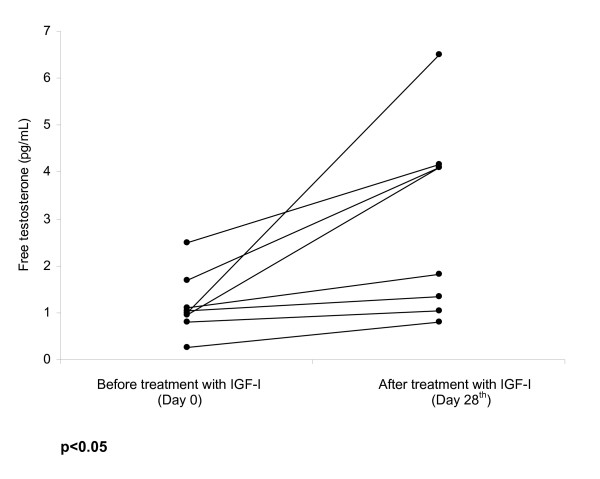
Testosterone levels before and after IGF-treatment in rats with testicular atrophy (AT+IGF) (p < 0.05).

Another interesting result was that comparing data from before and after treatment testosterone concentrations were increased (p < 0.05 before and after treatment) in all animals treated with IGF-I (Figure 4).

### Serum levels of IGF-I and IGFBPs expression

At the time of sacrifice (day 29^th^), 14–16 hours after the last IGF-I injection, surprisingly serum levels of IGF-I did not increase in rats with testicular atrophy treated with IGF-I (AT+IGF) (CO: 2257 ± 183 ng/ml; AT: 2209 ± 91 ng/ml; AT+IGF-I: 1910 ± 76 ng/ml).

**Figure 5:**
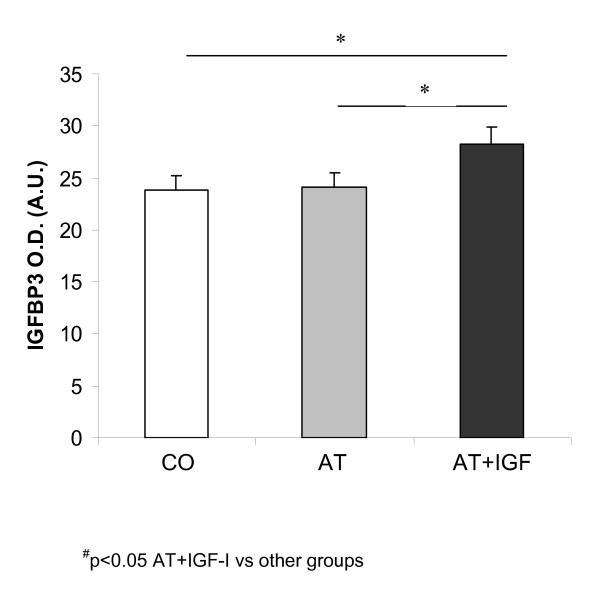
Plasma IGFBP-3 Western Blot expression (in arbitrary units of optic density) in the three experimental groups (CO, AT and AT+IGF);

The main IGFBPs were determined by Western-ligand-blot, showing changes in the pattern of IGFBPs with a significant increment of IGFBP3 in rats with testicular atrophy treated with IGF-I (Arbitrary Units, CO: 23.77 ± 0.48, AT: 23.01 ± 0.95, AT+IGF: 28.24 ± 1.11) (see Figure 5). An increase of IGFBP 1 and 2 (AU, CO: 3.31 ± 0.68; AT: 2.90 ± 0.78; AT+IGF: 6.47 ± 1.74) was also observed but it did not reach statistical significance.

### PHGPx activity in testicular homogenates

The values of PHGPx, an antioxidant enzyme, showed a significant increase in the AT+IGF group with regard to untreated animals with testicular atrophy (CO: 6 ± 0.2 IU/mg of protein; AT: 5 ± 0.3 IU/mg of protein; AT+IGF: 8 ± 0.7 IU/mg of protein), showing an antioxidant effect of IGF-I therapy on testes.

## Discussion

Insulin-like growth factor-I reverted testicular atrophy in rats with advanced cirrhosis, condition with "IGF-I deficiency" [1]. A question arises as to whether the beneficial effects of IGF-I on testicular atrophy are derived from the direct action of IGF-I or these effects are an epiphenomenon secondary to the general improvement induced by IGF-I in cirrhotic animals [1,5-8]. The direct action of IGF-I would allow us to suggest this therapy for other conditions with testicular damage.

In order to clarify this point, we employed another experimental model with a testicular atrophy and gonadal insufficiency induced by local ischemia for investigating the effect of IGF-I on testes in animals with neither hepatic disease nor malnutrition. However, testicular damages caused in this model were more severe than those found associated to advanced liver cirrhosis.

The animals included in the present study were shown to display a dramatic testicular damage manifested by a variety of histopathological abnormalities which include a reduction of testicular size, congestion and severe edema, alterations in tubular diameters, complete loss of the germinal line and total reduction of cellular proliferation as it has been previously reported [15-17]. These alterations could resemble in human pathology only those found in untreated testicular torsion [18].

In this assay, IGF-I treatment (at low doses) induced a little and partial improvement of testicular histology, increasing cellular proliferation and transferrin expression. In addition, IGF-I therapy increased serum testosterone levels (p < 0.05) (see Figure 4). The found results evidence a direct effect of IGF-I on testes but more interestingly show the difficulty to increase IGF-I serum levels and its availability using the reported low doses. These low doses were chosen in order to avoid hypoglucemia and other side effects.

Consistent with these findings, it has been reported that IGF-I exerts potent trophic and hormonogenetic effects on testis [1] and it is a critical autocrine and/or paracrine factor in the control of adult Leydig cells numbers and function and luteinizing hormone acts in part through IGF-I to stimulate proliferative activity [19]. The absence of IGF-I secretion in male GHR-KO mice is associated with a decreased plasma testosterone response to LH treatment and with a reduction in the number of testicular LH receptors [20].

In mouse, IGF-I gene null homozygous mutation led to a reduced testicular size, decrease of testosterone production and infertility [21]. It has also been reported that FSH-induced aromatization of androgens to estrogens in rat Sertoli cells in culture is markedly inhibited by IGF-I treatment through the reduction of aromatase mRNA [22]. Of interest, the recovery of transferrin expression in Sertoli cells observed in our study suggests a role of IGF-I in maintaining the integrity of the hematotesticular barrier [23] and consequently on cellular proliferation in some tubuli. Transferrin expresion by Sertoli cells is considered as a good marker of the testicular barrier integrity. This beneficial effect of IGF-I is in agreement with previous findings in animals with liver cirrhosis [1,24]. Since Sertoli cell factors influence on the Leydig cell function the improvement induced by IGF-I therapy on Sertoli cell may explain, at least partially, the found increase of testosterone.

Systemic IGF-I plays a major modulatory role in testicular endocrine function [20]. Interestingly, in the present work plasma levels of IGF-I did not increase in rats with testicular atrophy treated with IGF-I. Since the half-life for IGF-I is estimated in 14–22 hours [24,25], we propose that the hormone could be proteolyzed [26]. Similar findings have been observed administrating these low doses of IGF-I to animals with normal liver function. IGF-I is degraded within the endosomal apparatus as a consequence of receptor-mediated endocytosis [26]. These molecular mechanisms could be involved in the close regulation of circulating levels of IGF-I. Futher aditional studies could be necesary to understand better IGF-I regulation.

In addition, the availability of administered IGF-I seems to be reduced in this study since IGFBP-3 expression is increased after the IGF-I treatment (see Figure 5). Thus the exogenous supplementation of IGF-I induces an increase in the IGFBP-3 expression, which reduces the bioavailability of the administered hormone [24]. The ratio IGF-I/IGFBP3 is considered a marker of the IGF-I bioavailability [14,24], and normal spermatogenesis and perhaps also steroidogenesis are dependent on the actions of sufficient concentrations of unbound IGF-I [21,23,27,28].

The explanation for these results could be that this experimental model (testicular atrophy by hypoxia) is not an "IGF-I deficiency" condition. In this work IGF-I therapy induces some but little effects on testes, because the mechanisms of control of this hormone are closely regulated. The excess of IGF-I is down-regulated by the increase in IGFBP-3 and probably by the proteolytic metabolism of this hormone, because IGF-I plasma levels are not increased in IGF-I treated animals. These results suggest that the unique therapeutical possibility of IGF-I administration seem to be in conditions "with IGF-I deficiency" (as it is common with other hormones) although it is possible to see a direct action of IGF-I on testes in all conditions.

## Conclusion

In conclusion, the therapy with IGF-I is effective only in conditions of IGF-I deficiency, recovering testicular atrophy as it has been shown in advanced cirrhosis [1,23] or in Laron Syndrome [4]. In conditions of testicular damage where there is no IGF-I deficiency the exogenous administration of this hormone is buffered inducing only a little improvement on testicular histology and function.

The reported results in this work allow us to rule out the IGF-I supplementation as a therapeutic strategy in conditions without "IGF-I deficiency".

## List of abbreviations

IGF-I, insulin-like growth factor I; PCNA, nuclear antigen of cellular proliferation; CO, control healthy group; AT untreated rats with testicular atrophy induced by hypoxia; AT + IGF, IGF-I treated rats with testicular atrophy; GSHPx, glutathione peroxidase activity; IGFBP3, Insulin-like growth factor-I binding protein.

## Competing interests

The author(s) declare that they have no competing interests.

## Authors' contributions

FDC carried out the experimental model, the "in vivo" treatment, analytical determinations. ICC carried out the design, experimental model, histological analysis, "in vivo" treatment and coordination. MGF performed the analytical determinations, the statistical analysis and RIAs. JEP carried out the experimental model, in vitro treatment, bibliographic revision. MDS carried out the Immunohistochemical assays. ADC carried out the analytical determinations. MAAM participated in the "in vivo" assay and RIAs. CRB participated in the analytical determinations. SGB carried out the Revision. All authors read and approved the final manuscript.

## Pre-publication history

The pre-publication history for this paper can be accessed here:



## Figures and Tables

**Table 1:** Bioch emical data before and after IGF-I treatment (at low doses) in the three experimental groups.

**Parameters**		**Control rats **(CO, n = 10)	**Untreated AT rats **(AT, n = 10)	**AT rats treated with IGF-I **(AT+IGF, n = 10)
Albumin (g/dL)	Before	3.96 ± 0.16	3.60 ± 0.30	3.54 ± 0.30
	After	4.16 ± 0.24	4.04 ± 0.17	4.11 ± 0.27

Cholesterol (mg/dL)	Before	63 ± 6	64 ± 13	62 ± 12
	After	55 ± 9	54 ± 7	52 ± 7

Creatinine (mg/dL)	Before	0.61 ± 0.07	0.61 ± 0.06	0.68 ± 0.20
	After	0.79 ± 0.15	0.79 ± 0.08	0.71 ± 0.11

Alkaline phosphatase (UI/L)	Before	108 ± 22	127 ± 31	97 ± 27
	After	75 ± 15	84 ± 12	69 ± 14

Glucose (mmol/dL)	Before	9.3 ± 1.1	7.9 ± 1.9	9.0 ± 1.4
	After	11.1 ± 1.2	8.6 ± 0.8	8.2 ± 1.1

Total protein (g/dL)	Before	7.06 ± 0.29	6.32 ± 0.46	6.54 ± 0.61
	After	7.17 ± 0.31	6.90 ± 0.37	7.04 ± 0.42

Urea (mg/dL)	Before	35.6 ± 4.3	51.5 ± 7.2	48.1 ± 8.5
	After	37.4 ± 2.9	49.9 ± 9.0	41.2 ± 5.3

m ± SEM; n = 10, p = ns

**Table 2:** Parameters of testicular size and weight in the three experimental groups (day 29^th^).

	**Healthy Control rats **(CO, n = 10)	**Untreated AT rats **(CI, n = 10)	**AT rats treated with IGF-I **(CI+IGF, n = 10)
**Left teste weight·bw**^-1^	3.87·10^-3 ^± 1.28·10^-4^	2.75·10^-3 ^± 2.35·10^-4 ^^***^	3.06·10^-3 ^± 2.31·10^-4φ^
			
**External Testicular Diameters **(mm):			
• **Longitudinal**	16.05 ± 0.20	12.58 ± 0.22 ^***^	14.47 ± 0.30 ^** ^^&&^
• **Transversal**	7.20 ± 0.17	4.98 ± 0.37 ^***^	5.69 ± 0.22 ^**&&^

m ± SEM; ^*^p < 0.05 *vs *CO; ^**^p < 0.01 *vs *CO; ^***^p < 0.001 *vs *CO; φ p < 0.07 AT *vs *AT+IGF; ^&&^p < 0.01 AT *vs *AT+IGF; ^&^p < 0.05 AT vs AT+IGF

**Table 3:** Cellular analysis: 30 seminiferous tubuli were examined in each preparation. The table summarizes the number of tubuli in each category: category I = only Sertoli's cells; category II = I+espermatids; category III = II+espermatocytes; category IV= all types of cell but with some alterations; category V= all types of cells with normal features.

**Category**	I (number of tubules)	II (number of tubules)	III (number of tubules)	IV (number of tubules)	V (number of tubules)
**Controls (CO) **(n = 10) × 30 tubuli	0	0	0	15	285
**Rats with testicular atrophy (AT) **(n = 10) × 30 tubuli	120	62	44	12	62
**IGF-treated AT rats (AT+IGF) ** (n = 10) × 30 tubuli	25	25	56	81	113

Statistical analysis	p < 0.001 AT vs other groups	p < 0.001 AT vs CO p < 0.01 AT vs AT+IGF	p < 0.05 AT vs CO and AT+IGF vs CO	p < 0.001 AT+IGF vs CO and AT+IGF vs AT	p < 0.01 AT vs CO p < 0.05 AT vs AT+IGF p < 0.05 AT+IGF vs CO

**Table 4:** The pituitary-gonadal axis (on day 29^th^) in the three experimental groups.

	**Healthy Control rats (CO, n = 10)**	**Untreated rats with testicular atrophy (AT, n = 10)**	**IGF-I-treated AT rats (AT+IGF, n = 10)**
Total Testosterone (ng/dL)	88.10 ± 23.66	59.68 ± 19.70^*^	71.17 ± 15.86^#^
Free Testosterone (pg/mL)	7.35 ± 2.91	0.84 ± 0.67^***^	2.88 ± 1.45^** ^^&^
Estradiol (pg/mL)	8.20 ± 1.84	9.86 ± 3.35	9.93 ± 1.37
Estradiol/Total Testosterone	10.33 ± 7.11	61.47 ± 10.77	17.78 ± 13.92
LH (ng/mL)	2.16 ± 0.15	2.73 ± 0.36	3.28 ± 0.51^*^
FSH (ng/mL)	12.86 ± 1.20	17.80 ± 0.82^*^	20.58 ± 1.13^** ^^&^

m ± SEM; ^*^p < 0.05, ^**^p < 0.01, ^***^p < 0.001 CO vs other groups; ^&^p < 0.05 AT vs AT+IGF; ^#^p = 0.06 AT vs AT+IGF
